# Desiccation tolerance in bryophytes: The dehydration and rehydration transcriptomes in the desiccation-tolerant bryophyte *Bryum argenteum*

**DOI:** 10.1038/s41598-017-07297-3

**Published:** 2017-08-08

**Authors:** Bei Gao, Xiaoshuang Li, Daoyuan Zhang, Yuqing Liang, Honglan Yang, Moxian Chen, Yuanming Zhang, Jianhua Zhang, Andrew J. Wood

**Affiliations:** 1 Key Laboratory of Biogeography and Bioresource in Arid Land, Xinjiang Institute of Ecology and Geography, Chinese Academy of Sciences, Urumqi, 830011 China; 2 School of Life Sciences and State Key Laboratory of Agrobiotechnology, The Chinese University of Hong Kong, Hong Kong, China; 3 University of Chinese Academy of Sciences, Beijing, 100049 China; 4 Department of Plant Biology, Southern Illinois University-Carbondale, Carbondale, IL 62901-6509 USA

## Abstract

The desiccation tolerant bryophyte *Bryum argenteum* is an important component of desert biological soil crusts (BSCs) and is emerging as a model system for studying vegetative desiccation tolerance. Here we present and analyze the hydration-dehydration-rehydration transcriptomes in *B. argenteum* to establish a desiccation-tolerance transcriptomic atlas. *B. argenteum* gametophores representing five different hydration stages (hydrated (H0), dehydrated for 2 h (D2), 24 h (D24), then rehydrated for 2 h (R2) and 48 h (R48)), were sampled for transcriptome analyses. Illumina high throughput RNA-Seq technology was employed and generated more than 488.46 million reads. An in-house *de novo* transcriptome assembly optimization pipeline based on Trinity assembler was developed to obtain a reference Hydration-Dehydration-Rehydration (H-D-R) transcriptome comprising of 76,206 transcripts, with an N50 of 2,016 bp and average length of 1,222 bp. Comprehensive transcription factor (TF) annotation discovered 978 TFs in 62 families, among which 404 TFs within 40 families were differentially expressed upon dehydration-rehydration. Pfam term enrichment analysis revealed 172 protein families/domains were significantly associated with the H-D-R cycle and confirmed early rehydration (i.e. the R2 stage) as exhibiting the maximum stress-induced changes in gene expression.

## Introduction

The desiccation tolerant moss *Bryum argenteum* is emerging as an important model organism for understanding the molecular, structural and ecological aspects of vegetative desiccation tolerance in plants^1–4^, and is an important component of the biological soil crusts found in Northwestern China^5^. Desiccation tolerant (DT) mosses have the ability to dry completely (i.e. equilibrate their internal water potential to extremely dry air) and resume normal function upon rehydration^6^. DT is a common phenotype in bryophytes and more than 200 mosses have been experimentally verified to be desiccation tolerant^7^. Biological soil crusts are frequently subjected to cyclical desiccation-rehydration events and mosses such as *B. argenteum* have evolved remarkable constitutive and inducible mechanisms of desiccation tolerance in order to survive in these arid desert environments^2, 8, 9^.

Extensive research in the DT moss *Syntrichia ruralis* (=*Tortula ruralis*) has focused upon transcripts either stably maintained in desiccated tissues^10–13^ or preferentially expressed in rehydrated tissues^14, 15^. DT mosses are postulated to survive desiccation in part by employing a constitutive protection system and an active rehydration induced recovery mechanism^16^ within the context of robust structural and anatomical features^17, 18^. In response to drying, mRNA transcripts are sequestered in messenger ribonucleoprotien particles (mRNPs) and stably maintained in desiccated tissues^13^. Upon rehydration, these masked transcripts are preferentially selected and translated through activation of a repair-based mechanism. Next generation Illumina sequencing has been a powerful tool for gene identification and quantification, and a number of moss transcriptomes have been generated and characterized from hydrated and desiccated gametophores of the mosses *S. ruralis* ^19^, *Physcomitrella patens* ^20^, *B. argentuem* ^1^ and *Syntrichia caninvervis* ^21^. To better understand the mechanistic details of desiccation tolerance, recent transcriptomic analyses have emphasized the progressive dehydration and rehydration of moss vegetative tissues which is referred to as the Hydration-Dehydration-Rehydration (H-D-R) cycle.

Previously, our research group generated a *de novo* transcriptome for *B. argenteum* and analyzed digital gene expression (DGE) comparing three hydration stages (i.e., desiccated, 2 h post-rehydration and 24 h post-rehydration), in which neither hydration control nor biological replicates were conducted^1^. Furthermore, the sequencing depth of DGE analyses revealed to be not sufficient to quantify gene expressions accurately^1^. In this study, we generated an optimized and robust transcriptome assembly and analyzed gene expression (DGE) by comparing an expanded number of hydration stages for cultured *B. argenteum* gametophores: hydrated, dehydrated 2 h, dehydrated 24 h, rehydrated 2 h and rehydrated 48 h. This study compares transcript abundance during dehydration and rehydration. The data presented will provide greater insight into gene expression as plant tissues dehydrate/rehydrate and reveal molecular alterations associated with vegetative desiccation tolerance.

## Results and Discussion

### Drying and rehydration of de-hardened cultured *B. argenteum* gametophytes

Cultured *B. argenteum* gametophores were air-dried and the relative water content (RWC) decreased drastically to less than 2% after 4 h of desiccation, and 10 min after rehydration RWC recovered to more than 90% (Supplementary Fig. S1). Drying rates can be very rapid in exposed habitats but clump architecture in the BSCs can significantly slow water loss^22^. The cultured *B. argenteum* gametophytes have lost the compact clump architecture through which mosses can acquire rudimentary control over drying rate. Therefore, ambient RH was responsible for the drying rate as well as the eventual depth of desiccation. An important distinction between field-collected plant materials and the cultured plants used in this study is the physiological process of de-hardening^23^. The cultured material was de-acclimated for more than 7 days which removes the effects of the field thereby minimizing aspects of induced desiccation tolerance^7^. Mosses such as *B. argenteum* are fully desiccation tolerant and can survive desiccation without pre-treatment.

### Transcriptome *de novo* assembly, refinement and quality assessment

For non-model organisms without a reference genome, massive Illumina short-read sequencing, in conjunction with the efficient *de novo* transcriptome assembly, has become a flexible and robust method to generate a reference transcriptome, with sufficient depth coverage for subsequent differential gene expression analysis^24–26^. RNA-Seq libraries were prepared from *B. argenteum* gametophores of the well-hydrated (H0), 2 h post dehydration (D2), 24 h post dehydration (D24), 2 h post rehydration following desiccation (R2) and 48 h post rehydration (R48), representing five different hydration phases. A total of 488,456,309 paired-end reads (100 bp) were obtained by sequencing libraries on the Illumina HiSeq. 2500 platform (Supplementary Fig. S2). Preprocessing of the raw reads involved adapter/primer sequence triming and removal of low-quality reads. Clean reads, together with the Illumina reads previously generated^1^, were assembled *de novo* into a reference *B. argenteum* H-D-R transcriptome using the Trinity pipeline^24, 25^. Publically available transcriptome sequence data of *B. argenteum* generated by the 1000 plants (i.e. 1KP) project^27^ was not incorporated as the dataset was comparatively small and was obtained from plants grown in a vastly different habitat.

The resulting “Bryum_all” transcriptome, with 260,914 assembled transcript contigs (longer than 200 bp), N50 of 1,671 bp and average length of 943 bp, was subsequently subjected to a three-step refinement procedure to obtain an optimized transcriptome before the downstream differential expression analysis (Supplementary Fig. S2 and Table 1). The Trinity “long_isoform” selected the longest contigs from each Butterfly “gene” as described in the previous transcriptome assembly for *Eleusine indica* ^28^ and *Youngia japonica* ^29^. The decline of N50 and average length for the “long_isoform” dataset might be explained by the overrepresentation and higher degree of redundancy for longer genes, which contained more alternatively spliced isoforms. Mapping the cleaned reads back to the contigs, those likely misassembled contigs not well supported by reads, were filtered out based on abundance estimation (FPKM < 0.5), and 80,549 transcripts (denoted as “Bryum_filter”) were retained for further processing. In order to remove redundant and/or highly similar contigs, the filtered transcripts were then clustered at a sequence identity threshold of 98%. The resulting “Bryum_final” transcriptome contained 76,206 transcripts, with N50 of 2,016 bp and average contig length of 1,222 bp (Table 1). The refined “Bryum_final” transcriptome was used for all the subsequent analyses, including functional annotation and transcript abundance alteration analyses.

**Table 1:** Assembly, reads mapping and quality evaluation statistics for *Bryum argenteum* transcriptomes at each step of refinement.

Parameters	Bryum_all	Long_isoform	Bryum_filter	Bryum_final
No. of transcripts	260,914	215,754	80,549	76,206
Transcriptome size (Mb)	246.0	168.5	98.5	93.1
N50 (bp)	1,671	1,260	2,012	2,016
Average_length (bp)	943	781	1,223	1,222
Median_length (bp)	493	431	794	792
Minimum_length (bp)	224	224	224	224
Maximum_length (bp)	18,771	18,771	18,771	18,771
Total_mapped_reads (%)	90.03	85.96	84.90	84.67
Uniquely mapped reads (%)	52.13	69.62	68.78	74.38
TransRate-Assembly Score	0.23	0.25	0.32	0.36

To investigate the efficacy of transcriptome refinement, all the reads used for transcriptome assembly were mapped back to the *B. argenteum* transcriptomes generated for each step of the optimization (Table 1). Although the number of transcripts decreased drastically following the transcriptome refinement, only the “long_isoform” selection significantly decreased the reads mapping ratio, no further reduction of reads mapping ratio (approximately 85%) were observed upon the following steps of refinement. This indicated retention of most of the authentic transcripts throughout the optimization process, and the quality of the final transcriptome also increased in terms of N50 and average length (Table 1).

To further evaluate the quality of the “Bryum_final” transcriptome, a full-length transcript analysis was performed by aligning transcripts to the SwissProt database using BLASTX^25^. As a result, 63.9% of the transcripts with hits in SwissProt database were represented by nearly full-length transcripts, having more than 70% alignment coverage, and 78.2% of proteins demonstrated more than 50% alignment coverage (Supplementary Table S1). Completeness of the “Bryum_final” transcriptome assembly was then assessed by searching the transcripts against the 357 *A. thaliana* proteins that are conserved as single copy genes across all eukaryotes (i.e. ultra-conservedorthologs, UCOS, http://compgenomics.ucdavis.edu/compositae_reference.php) using BLASTX with an e-value cutoff of 1e-6^30^. As a result, the Bryum_final transcriptome dataset contained putative homologs of 355 UCOS genes, implying that the final refined transcriptome was largely complete. The TransRate package, a state-of-the-art reference free quality assessment tool of *de novo* transcriptome assemblies, calculates transcriptome assembly scores to assess the quality of transcriptome based upon reads mapping metrics and indicates the level of confidence in the final assembly^31^. The TransRate assembly score increased from 0.23 to 0.36 (Table 1), suggesting an overall transcriptome quality improvement by eliminating redundant and misassembled contigs.

### Annotation of the *Bryum argenteum* H-D-R transcriptome

Putative functional annotation information for the assembled *B. argenteum* transcripts were obtained by separate BLASTX searches (e-value ≤ 1e-5) against public protein databases including the plant division of NCBI-nr, the Swiss-Prot protein database, the *Arabidopsis thaliana* Araport11 database and the *Physcomitrella patens* COSMOSS v3.3 proteins (Table 2). A total of 42,852 (56.2%) transcripts acquired positive BLASTX hits from at least one of the four protein databases, among which 19,376 transcripts obtained high-quality Gene Ontology (GO) term information using Blast2GO as depicted in Supplementary Fig. S4. As part of Blast2GO pipeline, BLASTX searches against the NCBI-nr database simultaneously provided insight into the taxonomic distribution of the top hits (Supplementary Fig. S5). A total of 16,096 (41.6%) transcripts indicated top hits to sequences from the model moss *P. patens*, which is consistent with previously characterized moss transcriptomes^1, 21^. Among the 19,376 transcripts with GO terms assigned, the GO second level functional categories were summarized in Supplementary Fig. S4, which was similar with those previously reported^1^.

**Table 2:** Overview of the number of transcripts annotated with different databases.

**Databases**	NCBI-nr	SwissProt	Araport11	COSMOSS	Pfam	Transcription factors
**#Transcripts**	39,089	34,145	32,645	36,686	31,186	978

Aided by the BLASTX search results, the most likely coding sequences for the assembled transcripts were inferred or initially predicted and then translated into peptide sequences using the OrfPredictor algorithm (v3.0, standalone version)^32^. Finally, coding sequences for 55,648 transcripts (73% of the final transcripts) were predicted and translated into peptide sequences with a minimum length of 80 amino acids. These harvested peptides were then subjected to Pfam family/domain annotation using the HMMER software package^33^, and 31,186 peptides (56% of the peptides) were assigned with 4,618 distinctive Pfam family/domain information. Finally, the iTAK (v1.6) software package^34^ was used to excavate 978 TFs, which were further classified into 62 TF families.

### Characterization of global gene expression

**Figure 1:**
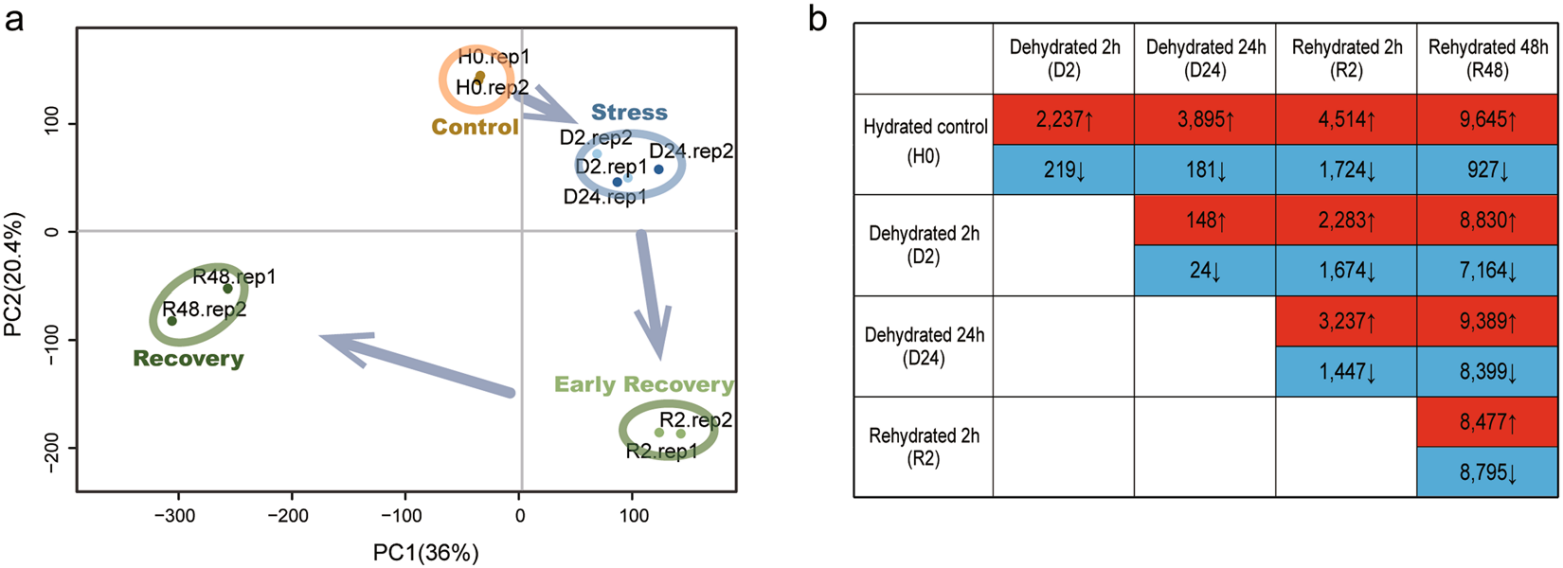
Global gene expression characteristics of *Bryum argenteum* during dehydration and rehydration. (**a**) Principal components analysis (PCA) of the biological replicates of desiccation-tolerant bryophyte *Bryum argenteum* subjected to well-watered conditions (control), after dehydration for 2 h and 24 h (stress) and recovery from dehydration for 2 h (early recovery) and 48 h (recovery). The arrows indicate the hydration process directions and transcriptomes from the same hydration stage were shown as dots of the same color. (**b**) Number of transcripts showing up- or down regulation during dehydration and rehydration.

The cleaned Illumina reads from samples of the five different hydration stages (H0, D2, D24, R2 and R48) were aligned back to the assembled Bryum_final transcripts separately (Supplementary Table S2). The number of reads aligned to each of the transcripts was obtained and used to estimate transcript abundances in units of FPKM using RSEM^35^. Principle component analysis (PCA) was conducted to illustrate the similar within-stage and different between-stage expression profiles among the H-D-R experimental groups, and simultaneously recapitulated the H-D-R continuum in the PCA space (Fig. 1a). As expected, the observed variability was associated with hydration status, suggesting that hydration (H0), dehydration (D2 and D24) and rehydration (R2 and R48) have distinctive gene expression patterns. Interestingly, all independent biological replicates for both drying stages (i.e. D2 and D24) were clustered together in the PCA space, suggesting their similar gene expression profiles and very limited transcript abundance changes upon extended dehydration, implying the sequestration of mRNAs upon desiccation. Whereas the two rehydration phases (i.e. R2 and R48) represented distinctive biological processes with substantial gene expression changes upon further rehydration (Fig. 1b). The dehydration process was conducted in dry air (ca 25% RH) which is analogous to the ambient desert atmosphere. Slow-drying (i.e. 3–6 hrs) is associated with vitrification and the preservation of biological molecules in the desiccated state^36, 37^.

The primary purpose of the transcriptome analyses was to obtain the significantly differentially abundant transcripts (SDATs) among different hydration stages (Fig. 1b). During the time course of dehydration, a total of 2,237 and 3,895 transcripts were more abundant, and only 219 and 181 transcripts were less abundant at 2 h and 24 h post dehydration, respectively. More than 90% of SDATs exhibited increased abundance upon desiccation and mRNA transcripts were stably maintained (minimum depletion) in desiccated tissues. And an additional 1,849 transcripts showed significant abundance elevations during the extended period of dehydration. Based on this scenario, we may suggest that upon “natural” desiccation the *B. argenteum* gametophytes might still be able to synthesize critical transcripts gradually pending for the repairing process upon rehydration, though no new mRNAs were recruited into the protein synthetic machinery during desiccation^14^. We suggested that the reserved rudimentary transcriptional activity during dehydration-desiccation may contribute to replenish the message pool to confer a quicker repair response upon rehydration based on a manner dependent on translational control. Compared with the dehydration process, more transcripts were differentially regulated upon rehydration. A total of 3,237 and 9,389 transcripts were more abundant and 1,447 and 8,399 were less abundant after 2 h and 48 h of rehydration, respectively. Though bryophytes rehydrate to reach morphologically full turgor almost instantaneously after the re-addition of water, substantial gene expression changes continued to be introduced after quite a long period of rehydration, suggesting the extended rehydration was associated with novel metabolic processes different from those introduced upon early rehydration.

### Gene expression patterns across different hydration stages

**Figure 2:**
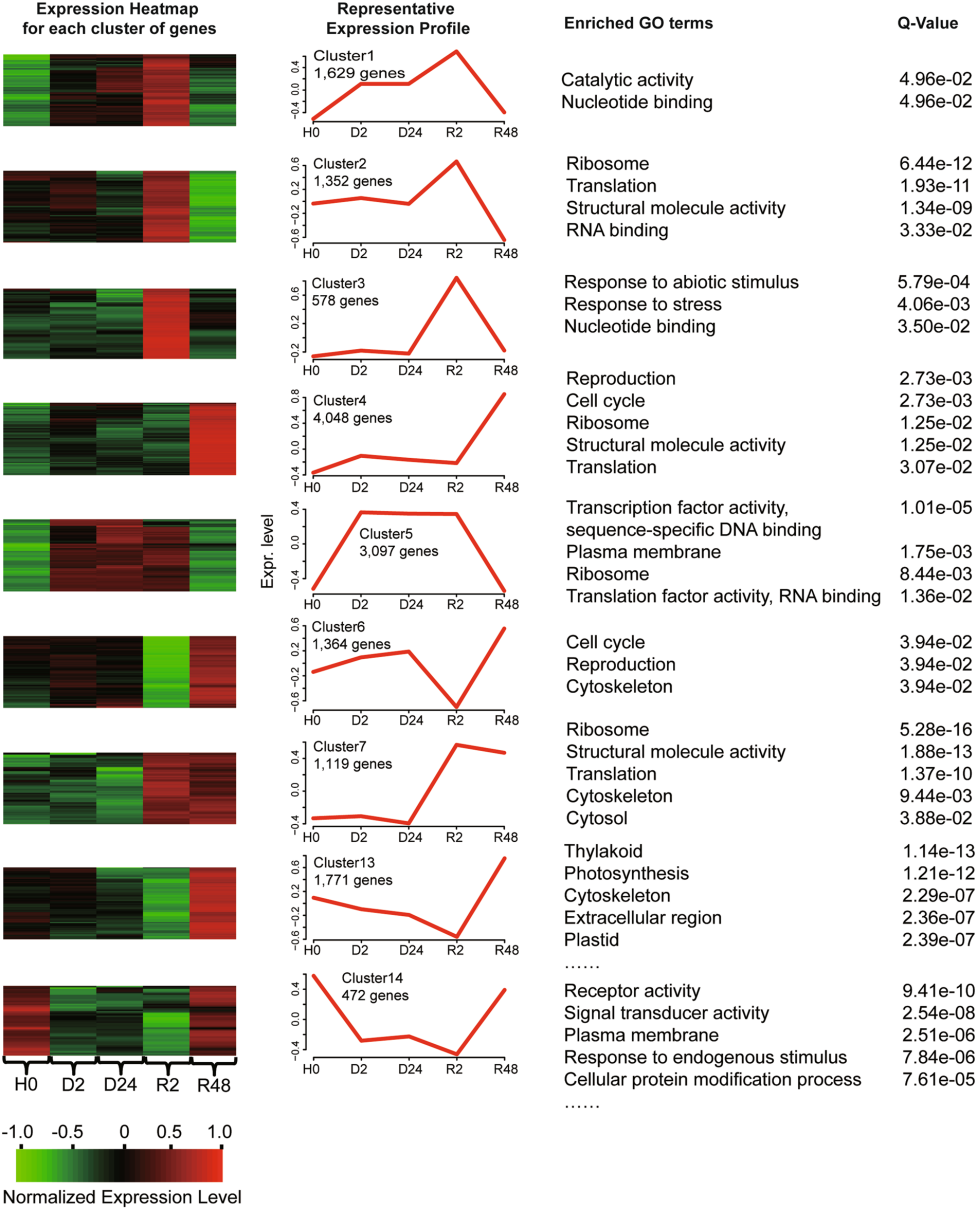
Clusters of genes showing representative expression patterns during dehydration and rehydration. All of the significantly differentially abundant transcripts (SDATs) were selected, then SOTA function in the clValid package was employed to classify these SDATs into 16 categories. The nine categories with significantly enriched GO terms were shown here. The top GO slim-plant terms and corresponding enrichment FDR values were shown in the right panel.

To elucidate the gene expression patterns during the time course of desiccation-rehydration, self-organization tree algorithm (SOTA) was employed to cluster all the 20,051 differentially abundant transcripts into 16 clusters, among which nine clusters exhibited significantly enriched GO terms (Fig. 2). A common signature for the first three clusters was maximal transcript abundance upon early rehydration. And the functionally enriched GO terms emphasized the importance of enhanced stress responsiveness and translational components. Abiotic stress responsive genes such as DNAJ heat shock proteins (TR3994|c0_g1_i1), temperature-induced lipocalins (TR82560|c0_g2_i1 and TR82560|c0_g3_i1), HSP90 (TR90818|c1_g2_i1), vacuolar ATP synthase (TR127463|c0_g1_i1) and heat shock factors (TR108063|c0_g1_i1 and TR116182|c0_g1_i2), were of peak abundance upon early rehydration and their abundances subsequently declined as rehydration progressed.

Consistent with the differential abundance analysis, “cluster 4” contained more than 4,000 transcripts that exhibited maximal abundance upon full recovery, and these transcripts were functionally enriched in “reproduction”, “cell cycle”, “ribosome”, “structural molecule activity” and “translation”. Transcripts in “cluster 7” contained the translational and structural genes which were more abundant in early rehydration and were (in many cases) retained upon full rehydration. A total of 3,097 transcripts captured within the “cluster 5” profile, of low abundance in H0 and R48, were more abundant upon early dehydration and maintained consistent transcript abundance level following desiccation and early rehydration. The enriched GO terms included “transcription activity”, “plasma membrane”, “ribosome” and “translation factor activity”. The abundance of transcripts in “cluster 5” were elevated upon early dehydration, maintained till early rehydration, and depleted following full recovery, suggesting rapid mobilization of transcriptional, membrane and translational components in de-hardened *B. argenteum* upon dehydration, as well as its powerful maintainence of these critical DT components in desiccated tissues.

Based on the “cluster 6” expression pattern and GO enrichment results, “reproduction” and “cell cycle” were significantly repressed upon early rehydration and strikingly enhanced following full recovery. Cluster 13 contained transcripts that were less abundant upon early rehydration but transcript abundances drastically increased following full recovery. Functional enrichment results demonstrated that the abundance of transcripts associated with photosynthesis was still low at the early stage of rehydration, but were significantly induced after 48 h post rehydration. As part of the homoiochlorophyllous mechanism of desiccation tolerance in *B. argenteum*, previous research using similar plant material failed to observe visible signs of damage in the chloroplast membranes nor a significant decrease in chlorophyll content upon desiccation^2^. Further, rehydration photosynthetic parameters were able to recover within 10 minutes independent of *de novo* photosynthetic proteins, whereas the long-term photosynthetic activity was demonstrated to rely upon protein re-synthesis^2^. These photosynthetic transcripts with increased abundance upon full rehydration consisted mostly of PSII light harvesting complex (LHCBs), as well as genes involved in PSI such as photosystem I light harvesting complex gene 3 (LHCA3, TR4081|c0_g1_i1), photosystem I subunit l (PSAL, TR27405|c0_g1_i1), Rubredoxin-like superfamily protein (TR141333|c0_g2_i1), cofactor assembly of complex C (CCB1, TR50015|c0_g1_i1) and photosystem II stability/assembly factor, chloroplast (HCF136, TR79996|c0_g4_i1). In contrast to the gene expression pattern in “cluster 5”, transcripts within “cluster 14” were of high abundance in both hydrated (H0) and fully rehydrated (R48) tissues but significantly less abundant during dehydration and early rehydration. Enriched functional categories of transcripts in “cluster 14” included the “receptor activity”, “signal transduction”, “kinase activity”, “cell communication”, “transporter activity” and “response to endogenous stimulus”.

### Functional and pathway enrichment analyses for SDATs

**Figure 3:**
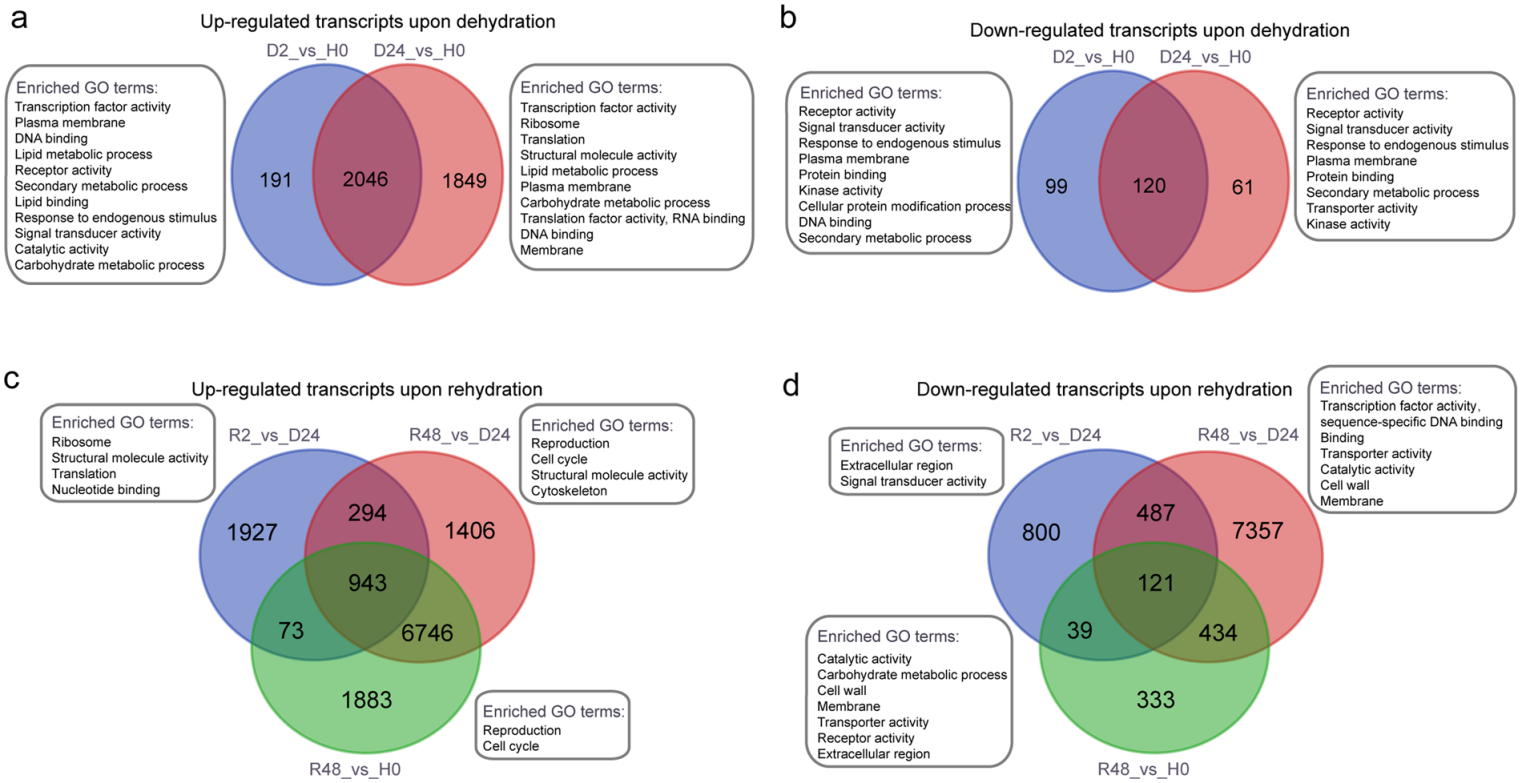
Differentially abundant transcripts and significantly enriched GO terms during dehydration (**a** and **b**) and rehydration (**c** and **d**). (**a** and **b)**, Number of transcripts with significantly elevated abundance (**a**) and decreased abundance (**b**) compared to hydrated control during dehydration with enriched GO-slim terms. **c** and **d**, Number of abundance elevated (**c**) and abundance decreased (**d**) transcripts compared to desiccated (D24) and hydrated control during rehydration with enriched GO-slim terms. H0: hydrated control, D2: dehydrated 2 h, D24: dehydrated 24 h, R2: rehydrated 2 h, R48: rehydrated 48 h.

To better understand the H-D-R hydration cycle, we performed five pairwise comparisons associated with dehydration and rehydration. The SDATs were used for GO and KEGG pathway enrichment analyses to elucidate the molecular mechanisms associated with the desiccation-rehydration continuum (Fig. 3). Significantly over-representative GO terms for transcripts with elevated abundance included “transcription factor activity”, “translation”, “ribosome” and “plasma membrane”. Whereas significantly enriched GO terms for abundance decreased SDATs during the H-D-R continuum included “receptor activity”, “signal transducer activity” and “response to endogenous stimulus”. Upon early dehydration, genes related to “transcription factor activity”, “plasma membrane”, “lipid metabolic process”, “signal transducer activity” and “carbohydrate metabolic process” were significantly elevated (Fig. 3a), suggesting the accumulation of transcripts associated with these metabolic processes. Unlike the well-established signaling pathways associated with water deficit responses in angiosperms, desiccation tolerant bryophytes rely on translational controls to mount a repairing response to survive desiccation^13^. We postulate that repair mechanisms based on translational control are pivotal for DT in bryophytes, but the transcription of some crucial transcripts would also be a critical event. This is especially true for de-acclimated tissues, as protection components were already in place for field-collected or hardened materials but could have been depleted after long-term culture^38^. Two 9-cis-epoxycarotenoid dioxygenase (NCED)-coding transcripts (TR51785|c0_g1_i1 and TR79202|c0_g1_i1) exhibited significantly increased abundance upon dehydration and their abundance decreased following full recovery, implying ABA might play a role in DT of *B. argenteum*. Earlier study has proposed that phospholipid-based signaling besides ABA is likely to play an important role in the acquisition of DT, and Phospholipase D (PLD, a phospholipid cleaving enzyme) has a role in tolerance induction as a secondary, intracellular messenger^39^. Transcripts encoding PLDs (such as TR111012|c0_g1_i1 and TR50019|c0_g1_i1) demonstrated elevated abundances upon early dehydration, maintained upon early rehydration and depleted following full recovery, whereas abundance of some PLD transcripts accumulated upon full recovery.

**Figure 4:**
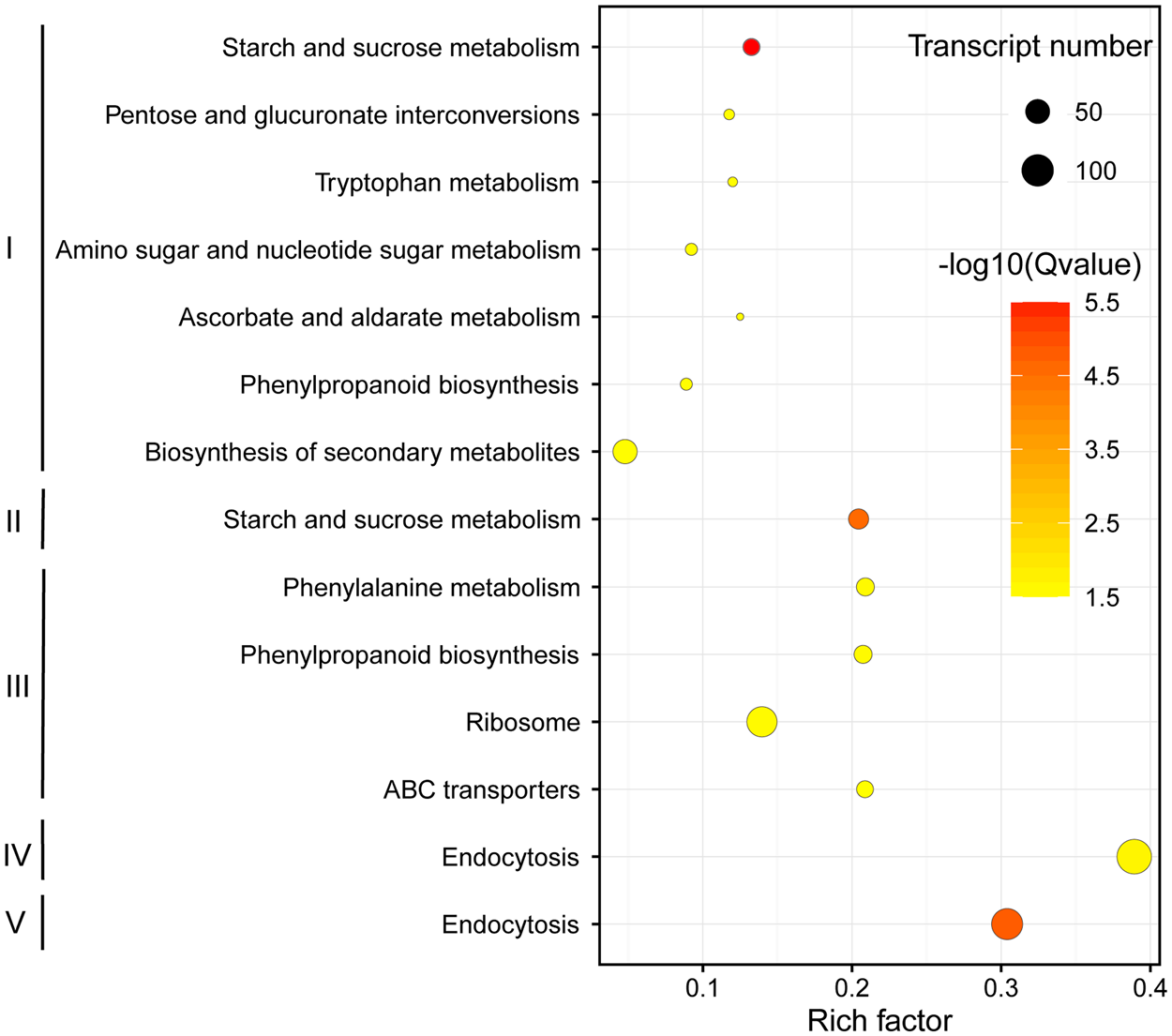
Scatterplot of all statistically enriched KEGG pathways for differentially expressed genes during dehydration and rehydration. The characters I, II, III, IV and V in the figure correspond to the comparisons D2_vs_H0, D24_vs_H0, R2_vs_D24, R48_vs_D24 and R48_vs_H0, respectively. Rich factor was the ratio of the number of SDATs to the total gene number in a specific pathway. The size and color of the dots represent the transcript numbers and the FDR corrected P-values, respectively.

To identify the metabolic pathways that were significantly altered by dehydration and rehydration, we used KEGG pathway analysis to uncover altered metabolic pathways. Significantly altered KEGG pathways were identified using a P-value based evaluation upon the hypergeometric distribution (Fig. 4). Upon early dehydration (Group I) and desiccation (Group II), transcripts related to “starch and sucrose metabolism” were significantly altered. Following early rehydration (Group III), the abundance of transcripts associated with “ribosome” changed significantly, suggesting the importance of translational regulation upon early rehydration. For full rehydration (Group IV and V), a number of transcripts with functions related to “endocytosis” exhibited significant abundance alterations. Coinciding with the GO enrichment results, the “starch and sucrose metabolism” pathway was differentially regulated at both D2 and D24 (Fig. 4), suggesting the potential accumulation of sucrose upon dehydration. Abundance of a transcript (TR87355|c0_g1_i1) encoding sucrose synthase (SuSy) increased drastically 2 h post dehydration. The non-reducing disaccharide trehalose is also a powerful protectant during desiccation, and able to confer desiccation tolerance to *Saccharomyces cerevisiae* ^40, 41^, but rarely accumulated by plants^42^ and its low contents in plants are insufficient to act as either a chaperone or energy source^43^. It is possible that sucrose in green plants plays the same role as trehalose in other organisms. Experiments with liposomes have shown that sucrose can protect them from desiccation-induced lateral phase separation^36^. Recent research has demonstrated that during desiccation trehalose could trigger autophagy to prevent programmed cell death (PCD) in the resurrection grass *Trigopon loliiformis* ^44^. It was also noticed the abundance of transcripts (TR141728|c0_g1_i1, TR118891|c0_g1_i1 and TR128507|c0_g1_i1) encoding trehalose-6-phosphate synthase (TPS) were more abundant upon desiccation and declined upon rehydration, whereas the precise role of trehalose within DT mechanisms remains elusive. Other enriched pathways upon early dehydration included the “amino sugar and nucleotide sugar metabolism”, “ascorbate and aldarate metabolism”, probably associated with the osmotic and anti-oxidative protection.

### Gene families associated with desiccation-rehydration

**Figure 5:**
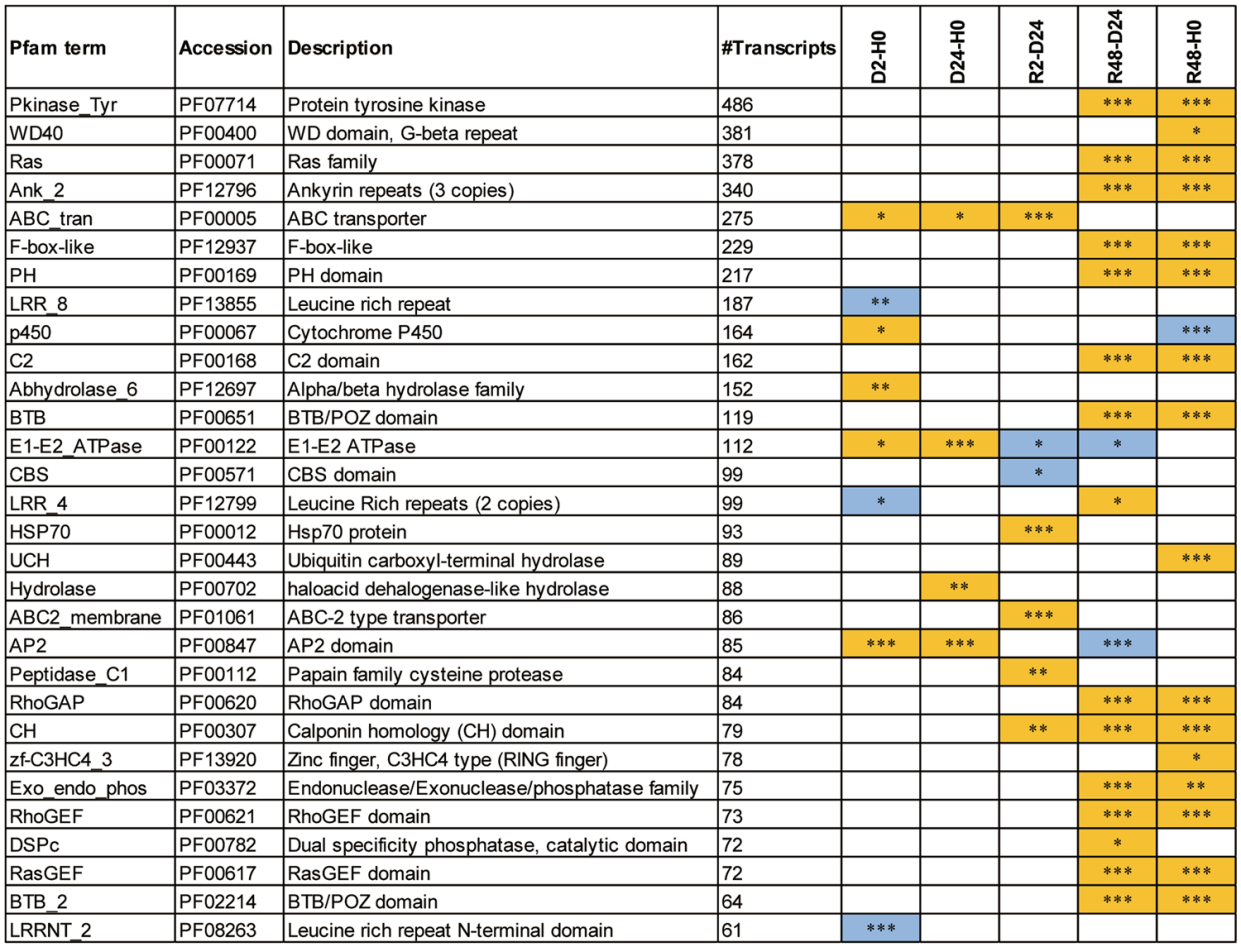
Protein family (Pfam) terms enriched for up- and down-regulated transcripts, upon dehydration, desiccation and rehydration. The yellow color indicates enriched up-regulated and blue down-regulated Pfam terms. Significance levels are marked by asterisks in the boxes (***FDR ≤ 0.001; **0.001 < FDR ≤ 0.01; *0.01 < FDR ≤ 0.05). The p-values were FDR corrected and cut-off was set at FDR ≤ 0.05.

**Figure 6:**
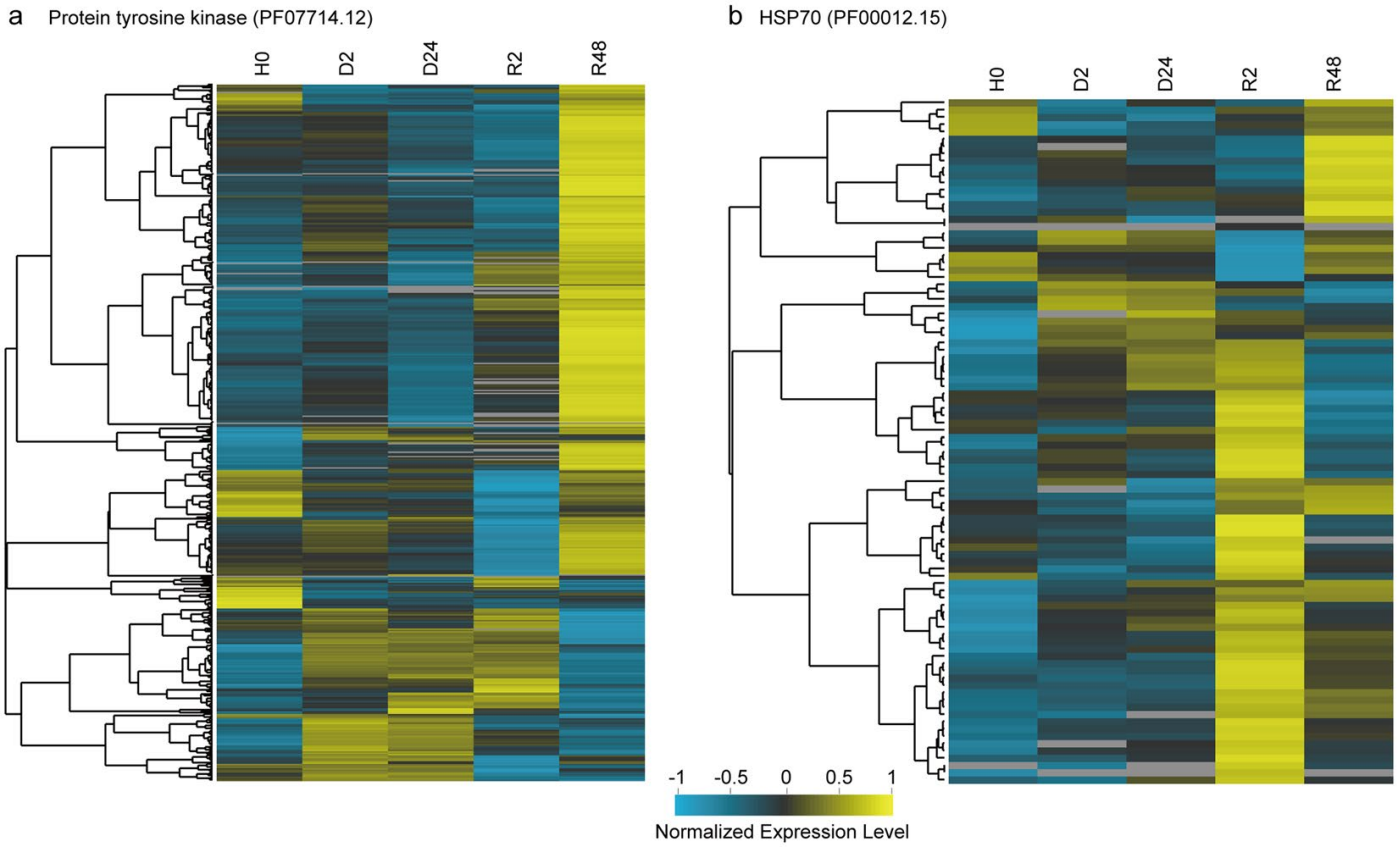
Heatmaps illustrating the expression profiles of the protein tyrosine kinase (**a**) and heat shock protein 70 family (**b**) transcripts upon dehydration and rehydration.

In accordance with the GO enrichment analysis, a total of 172 Pfam terms were enriched (FDR < 0.05) for the desiccation-rehydration response in *B. argenteum*, and the top 30 populous enriched Pfam terms were listed in Fig. 5. Pfam enrichment analysis revealed significant changes in Pfam terms including “Pkinase_tyr”, “WD40”, “Ras”, “Ank_2” and “ABC_tran”. These significantly abundance elevated Pfam terms contained the largest number of transcripts, ranging from 486 to 275, associated with multiple hydration stages. HSP70 family contained 93 transcripts and was correlated with significant abundance elevations specific to early rehydration. The LRR_8 revealed to be the largest Pfam term that was significantly less abundant upon early dehydration (Fig. 5). Taking a closer examination of the expression profiles of the Pkinase_tyr and HSP70 families, a number of Pkinase_tyr and HSP70 transcripts were significantly more abundant upon full rehydration, and quite a large number of HSP70 transcripts significantly accumulated upon early rehydration (Fig. 6). Furthermore, we examined the expression profiles of late embryogenesis abundant (LEA) group protein coding transcripts (Supplementary Fig. S6) as well as the HSP90, HSP20 and HSP33 family transcripts (Supplementary Fig. S7). The HSP90 transcripts exhibited similar expression patterns with HSP70. Impressively, the LEA protein-coding transcripts were mostly accumulated in desiccated tissues, maintained upon early rehydration and depleted following full recovery.

Proteomic analysis of *P. patens* in response to dehydration/rehydration identified 71 dehydration responsive proteins^45^. In the study by Wang *et al*., dehydrins (group 2 LEAs), heat shock protein 70 and HSP 70-like proteins were among the most abundant, up-regulated, dehydration stress-responsive proteins in *P. patens*; in addition, several protein kinases were up-regulated including PfkB-type carbohydrate kinase, Ser/Thr-specific protein kinase-like and Calcium dependent protein kinase-like (CDPK-like) proteins^45^. Subsequent proteomic analysis of *P. patens* also demonstrated that dehydrin, group 3 LEAs, HSP70, HSP70-2, HSP70-3 and HSP100 were among the most abundant, up-regulated, dehydration stress-responsive proteins^46^. Transcriptomic analysis of *P. patens* has demonstrated that dehydrin (group 2 LEA) transcripts are more abundant in dehydrated gametophytes^20, 47^, while transcripts encoding putative dehydrins, HSP and HSP-like proteins are more abundant in both slow-dried (SD) and rapid-dried (RD) *S. ruralis* ^19, 48^. Dehydration stress-accumulation of LEA, HSP and HSP-like gene products might be a common response of land plants in both vegetative and reproductive tissues^1, 12, 14, 18, 19, 36, 38^, and is postulated to be a key component of vegetative DT in bryophytes^18, 19^.

**Figure 7:**
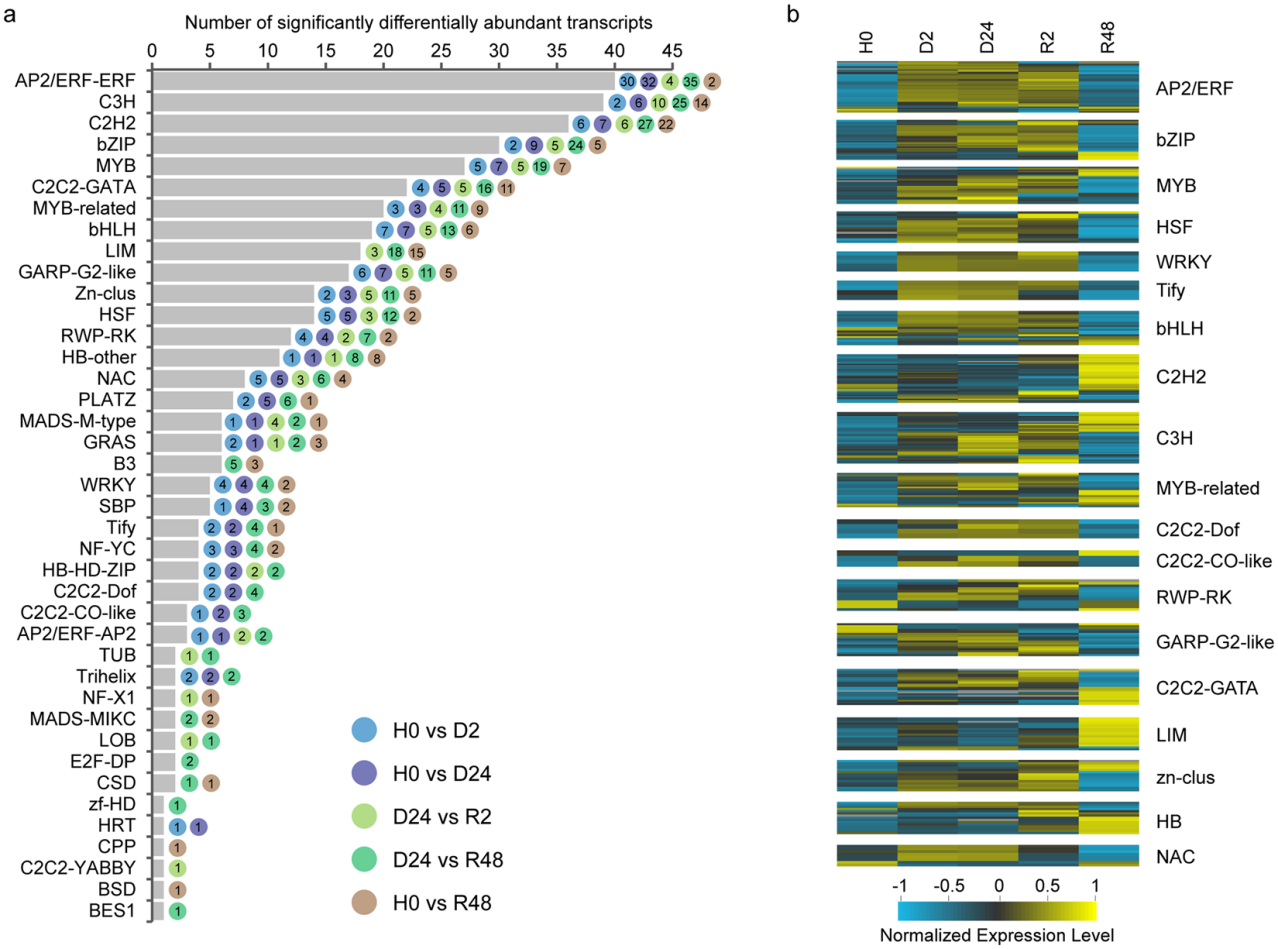
Abundance altered transcription factor (TF)-coding transcripts in *Bryum argenteum* during dehydration and rehydration. (**a**) A total of 404 TFs belonging to 40 TF family exhibited significantly differential abundance during dehydration and rehydration. The color code showed the differential abundance of transcripts belonging to a certain TF family for that hydration stage in comparison with H0 or D24. (**b**) Heatmaps illustrating the expression profiles of the significantly differentially abundant transcripts of interested TF families.

As a major part of gene expression regulatory components, a total of 978 TFs within 62 TF families were annotated, among which 404 TF-coding transcripts belonging to 40 TF families were differentially expressed during the time course of dehydration-rehydration (Fig. 7a). Among these TF families, 27, 23 and 35 families were associated with the dehydration, early rehydration and full recovery processes, respectively. Majority of the differentially abundant TF families were responsive to both dehydration and rehydration, and the LIM family, important transcriptional regulator for key phenylpropanoid pathway genes and lignin biosynthesis^49^, was specially associated with the rehydration process. And we also considered that the intermediate products for lignin biosynthesis could contribute to the cell wall constitutes though lignin is not present in bryophytes^50^. Heatmaps of abundance altered TFs were plotted to depict the transcript abundance profiles of each individual TF family (Fig. 7b). And based on their gene expression preferences, they could be primarily classified into three categories associated with “stress”, “development” and both^51^. Scrutiny of the differentially expressed TFs in AP2/ERF, MYB, HSF, WERK, Tify families demonstrated their preferential stress responsiveness, all of which have been classified as “first class” TF families responsive to stress or stimulus^51^. Most of these TF family members were more abundant upon early dehydration and maintained elevated abundance until early rehydration, but drastically decreased after full recovery (Fig. 7b), suggesting their critical regulatory roles in DT and early rehydration. We observed majority of TFs in the C2H2 and LIM families were predominantly more abundant at R48, suggesting a role in long-term recovery from desiccation. Several TF families, such as bZIP, bHLH, C3H, MYB-related, C2C2-GATA, HB and zn-clus, illustrated their dual functions in both “response to stress” and “developmental process”.

Transcriptomic analysis has also demonstrated the AP2/EREB TF transcripts are more abundant in dehydrated *P. patens* ^20, 52^, and our research group has characterized the AP2/ERF gene family in *S. caninervis* ^53^. Interestingly, though AP2/ERF gene family assembled and annotated in the DT moss *S. caninervis* transcriptome is less than 50% the size of either *A. thaliana* (80 sequences vs 176) or *P. patens* (80 sequences vs 171), in both *P. patens* and *S. caninervis* the ERF subfamily represents 90% of the AP2/ERF sequences (as compared to 77% for *A. thaliana*)^53^. APETALA2/Ethylene Responsive Factor (AP2/ERF) is a large family of plant transcription factors that play important roles in the control of plant metabolism and development as well as responses to various biotic and abiotic stresses^54^. Currently, ongoing research in our labs is aimed at evaluating the putative role of A-5 type DREB proteins in abiotic stress tolerance.

### qPCR validation for interested genes

**Figure 8:**
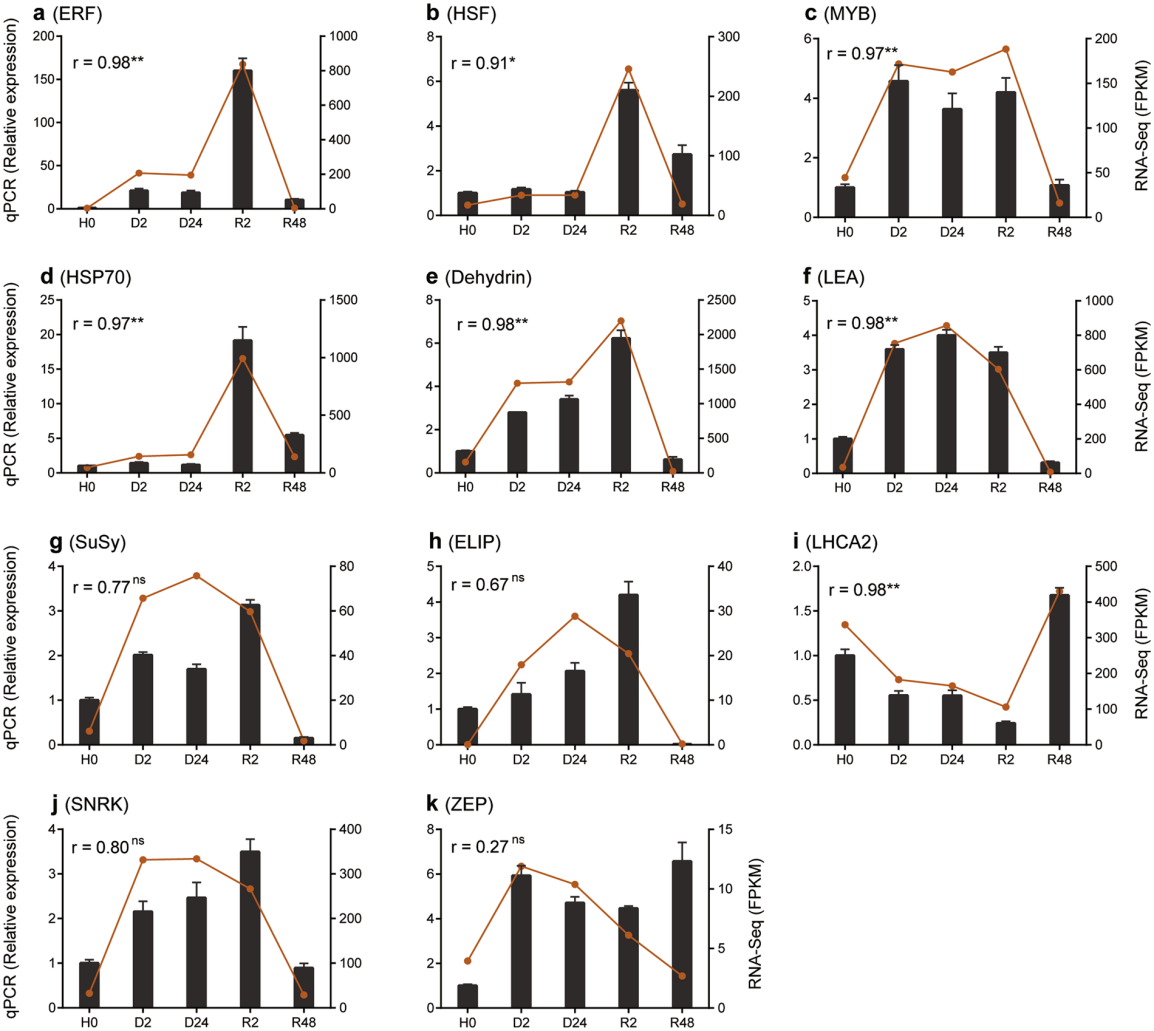
qPCR validation of interested genes in *Bryum argenteum*. Selected transcript abundance profiles were validated using quantitative real-time PCR (qPCR), including genes encoding ERF transcription factor (**a**), heat shock factor (HSF) (**b**), MYB transcription factor (**c**), heat shock protein 70 (**d**), dehydrin (**e**), late embryogenesis abundant (LEA)-like protein (**f**), sucrose synthase (**g**), early light induced protein (ELIP) (**h**), photosystem I light harvesting complex gene 2 (LHCA2) (**i**), protein kinase (SNRK) (**j**) and zeaxanthin epoxidase (ZEP) (**j**). qPCR quantitative gene expression data were shown as the mean ± SEM. Correlation of the expression levels evaluated using qPCR and RNA-Seq was statistically assessed by the calculation of Pearson correlation coefficient (r) and P-value were also indicated (**P-value < 0.005, *P-value < 0.05, ns: not significant or P-value > 0.05).

The expression profiles of 11 transcripts spanning different aspects of water deficit response were independently analyzed by qPCR (Fig. 8). Among these, the expression of MYB transcription factor (Fig. 8c), SNRK (Fig. 8j) and ZEP (Fig. 8k) were significantly elevated in dehydrated (i.e. D2 and D24) as compared to hydrated (H0) tissues. All of the analyzed transcripts, with the exception of LHCA2 (Fig. 8i) and ZEP (Fig. 8k), were significantly elevated in early rehydration (i.e. R2) as compared to both hydration (H0) and rehydration (R48). This independent qPCR evaluation demonstrated the reproducibility of the gene expression profiles derived from high throughput RNA-Seq quantification.

## Concluding Remarks

Desiccation tolerance is the ability of cells to dry completely and survive. Vegetative DT in bryophytes is a common phenotype and understanding the molecular basis of the phenotype is predicated upon expanding our inquiry to other exemplar species such as *B. argenteum*. Interestingly, *B. argenteum* is one of the first bryophytes experimentally determined to be DT^7, 55^. In this study, we generated an optimized *de novo* transcriptome assembly and analyzed the gene expression profiles of *B. argenteum* subjected to a detailed H-D-R cycle. Characterizing the biochemical and molecular alterations during dehydration & rehydration has been challenging since DT is influenced by a host of “experimental conditions”-from the speed & intensity of desiccation to the acclimation status of the plant material. In this study, we investigated the transcriptome of a fully DT moss, collected from the Gurbantunggut Desert and subsequently grown in tissue culture thereby allowed to deharden^7, 23^, dried slowly at 25% RH and then rehydrated. In this context, we can determine relative transcript abundance and evaluate the potential role these genes play in vegetative DT. Consistent with *S. ruralis*, transcripts associated with translation and repair accumulate during drying and are stably maintained during desiccation. Interestingly, transcripts encoding TFs also accumulate during drying even though transcription and translation are limited in dry plant cells. Our long-term interest in understanding mRNA stability now encompasses potentially sequestered TF transcripts. We postulate that sequestered TF transcripts encode proteins active upon rehydration (possibly in early dehydration) which are responsible for both repair and the re-establishment of phenotypes altered by de-acclimation. This information is a unique resource for understanding the gene expression profiles associated with changing cellular water content and provides an expanded molecular toolbox for evaluating the role(s) of transcriptional and translational gene control in vegetative desiccation tolerance in bryophytes.

## Materials and Methods

### Plant material

*Bryum argenteum* gametophores were originally collected from the Gurbantunggut Desert in Xinjiang, China. Gametophytes were cultured in 9 cm Petri dishes on solid Knop culture medium at 25 °C with a 16 h photoperiod (under cool white fluorescent light, ~4000 lux) in a climate chamber. The gametophore manipulations were carried out in a walk-in environmental control room. For experimental purposes, the well-hydrated gametophytes were harvested from Petri dishes, cleaned to get rid of excessive water and employed as the hydrated control sample (denoted as H0). *B. argenteum* gametophytes were air-dried in open Petri dish exposed to 25% RH at 25 °C for 2 h (denoted as D2) and 24 h (denoted as D24) to obtain early dehydrated and desiccated gametophores, respectively. This air-drying regime could result in the attainment of the gametophytes to lose more than 95% of their wet weight. Desiccated gametophytes (D24) were rehydrated for 2 h (denoted as R2) and 48 h (denoted as R48) with deionized water, representing the early recovery and fully rehydrated samples, respectively.

### RNA-Seq Library Preparation and Sequencing

Total RNAs were extracted from *B. argenteum* gametophytes with 2 biological replicates collected for each of the five different hydration stages (i.e. H0, D2, D24, R2 and R48). RNA-seq libraries were prepared using TruSeq RNA Sample Prep Kits (Illumina) according to the standard manufacturer’s protocol and multiplexed RNA-seq libraries were sequenced on the Illumina HiSeq. 2500 sequencing platform. All the Illumina sequencing reads of the RNA-Seq libraries have been deposited at the NCBI Sequence Read Archive (SRA) repository (http://www.ncbi.nlm.nih.gov/sra) with accession numbers of SRR3740898, SRR3740899, SRR3740900, SRR3740901, SRR3740912, SRR3740921, SRR3740922, SRR3740924, SRR3740929 and SRR3740930 under the BioProject PRJNA327617.

### Preprocessing of raw reads and *de novo* transcriptome assembly

Preprocessing of raw reads involved removal of adapter and primer contamination and Q20-quality trimming (removal of low-quality reads with average Phred quality score <20 and trimming of low-quality bases from the both ends of the reads) using Trimmomatic (v0.33)^56^. Read pairs where both reads were of at least 36 bp in length after this quality control procedure were retained for subsequent analyses. And all the cleaned reads were pooled for *de novo* assembly together with the previously sequenced Illumina reads (NCBI-SRA accession: SRR1763242) generated by our group previously^1^, so that the resulted transcriptome would represent the most comprehensive *B. argenteum* transcriptome to date. The Trinity software package (v2.0.6) was employed for efficient and robust *de novo* assembly of a reference transcriptome from the RNA-seq reads^24, 25^. Specifically, our *de novo* transcriptome assembly was performed with the command line parameters of “–normalize_max_read_cov 30 –min_kmer_cov 2 –min_contig_length 200”. And all the assembled transcript sequences were collectively denoted as the “Bryum_all” transcriptome.

### Refinement and quality assessment of transcriptome assembly

From the *de novo* assembled “Bryum_all” transcriptome, a “long_isoform” set was constructed by extracting the longest “isoform” from each Trinity Butterfly “gene” as described previously^28, 29^. In order to filter out transcriptional artifacts, misassembled and poorly supported transcripts, all cleaned reads were mapped back to the assembled transcripts using Bowtie2, followed by SAMtools usage to generate alignment file in .bam format^57, 58^. RSEM software was subsequently employed to calculate the abundance for each transcript in FPKM units (fragments per kilobase per transcript per million mapped reads), and only transcripts with equal to or bigger than 0.5 FPKM were retained for further processing^35^. CD-HIT suite, a clustering program based on similarity threshold, was employed to handle the issue of highly similar/redundant transcript contigs^59^. Specifically, CD-HIT-EST with a sequence similarity threshold of 0.98 and word size of eight was used to eliminate redundant transcript sequences. And the resulted non-redundant transcript sequence dataset, denoted as the “Bryum_final” transcriptome, was employed for all the subsequent bioinformatic analyses.

To evaluate the refinement process, reads were mapped back to transcripts at each stage of transcriptome refinement using Bowtie2 with the same parameters as described previously^60^. And percentage of the totally and uniquely mapped reads were reported by the use of SAMtools flagstat to evaluate the quality and completeness of the refined transciptomes^58^. For full-length transcript analysis, transcripts were compared against the manually curated and reviewed SwissProt protein database (http://www.uniprot.org/downloads) using parameters of “-evalue 1e-20 -num_threads 16 -max_target_seqs 1 -outfmt 6”, and then Perl script from the Trinity suite was used to examine the percentage of the Swiss-Prot target being aligned to the best-matching assembled transcript^25^. The TransRate (http://hibberdlab.com/transrate/index.html) package (v1.02), developed at the University of Cambridge and Oxford, was run on the read evidence mode to calculate the overall assembly score for each of the transcriptomes by mapping reads back to the contigs and inspecting the alignments^31^. The overall transcriptome assembly score ranges from 0 to 1 and an increased score is very likely to correspond to an assembly that is more biologically accurate.

### Functional annotation of the transcriptome

All the harvested “Bryum_final” transcripts were subjected to BLASTX search against the plant division of the NCBI-nr database (e-value ≤ 1e-5). The standalone perl script OrfPredictor (v2.3)^32^ and the BLASTX results were used to predict the coding frame and generate peptide translations for each of the transcripts. The resulting predicted polypeptide sequences were filtered with a minimum length of 80 amino acids. The deduced polypeptide sequences were then subjected to search against the Pfam-A (v27.0) for protein domain and family assignment using HMMER (v3.1b1)^33^. Transcription factors were classified using the stand-alone iTAK package (v1.6)^34^ according to the classification rules proposed by PlnTFDB^61^ and PlantTFDB^62^ databases.

Functional annotation and GO labels for each transcript was obtained using homolog search results followed by Blast2GO mapping and annotation processes^63^. All final transcripts were searched against the NCBI-nr database using BLASTX with an initial e-value threshold of 1e-5 and a maximum of top 20 hits per query sequence. Then the BLASTX output, generated in.xml format, was used for Blast2GO analysis to annotate the transcripts with GO terms describing biological processes (BP), molecular functions (MF) and cellular components (CC). GO annotations were retrieved and assigned to each transcript and filtered using the following cutoffs: e-value filter 1e-6, annotation cutoff 55, GO weight 5 and Hsp-Hit coverage cutoff 0. The resulting GO annotation was functionally classified using WEGO (http://wego.genomics.org.cn/)^64^ at specified GO hierarchical levels.

### Differential expression analysis

RNA-seq by expectation maximization (RSEM) package was used for transcript abundance estimation of the *de novo* assembled transcripts^35^. Reads from sequencing libraries of each hydration stage were mapped to the transcripts with default RSEM parameters using script “run_RSEM_align_n_estimate.pl”, followed by joining RSEM-estimated abundance values for each sample using the Perl script “merge_RSEM_frag_counts_single_table.pl”. Finally, differential abundance analysis was performed using the Perl script “run_DE_analysis.pl”, which involves the bioconductor package EdgeR in the R statistical environment^65^. Removal of transcripts with very low estimated counts (i.e. a minimum total number of 10 reads for the combined data set) was done prior to pairwise comparisons for each tissue pair using EdgeR. The differentially expressed transcripts were determined using FDR < 0.001 and absolute value of log2 (fold-change) >2 as threshold. All these Perl scripts above are bundled with the Trinity (v2.0.6) software suit^24, 25^.

### Gene Ontology and metabolic pathway enrichment analysis

BiNGO, a plugin in Cytoscape, was used to calculate the overrepresentation of GO terms using the hypergeometric test and Benjamini & Hochberg False discovery rate (FDR) correction^66^, with corrected P-value ≤ 0.05 as threshold of the significance level^67, 68^. And the collapsed “goslim_plant” was selected as name space to simplify the GO enrichment results. To identify the metabolic pathways involved in the desiccation-rehydration process, all the transcripts were subjected to BLASTX search against the *Physcomitrella patens* sequences downloaded from KOBAS (http://kobas.cbi.pku.edu.cn/). Local BLASTX search was performed with an initial e-value threshold of 1e-5 and the search results were filtered by keeping BLASTX hits showing at least 30% identity and a minimum aligned length of 80 amino acids. The filtered BLASTX results were submitted to KOBAS webserver^69^ to “annotate” the KEGG metabolic pathways for each transcript with an e-value cutoff of 1e-8. Hypergeometric test was subsequently performed to “identify” the differentially regulated pathways with a threshold of FDR ≤ 0.05 as the significance level.

### Analysis of gene expression patterns during desiccation-rehydration

Cluster analysis of the gene expression patterns was performed using Cluster 3.0^70^ and Java Treeview^71^ softwares. Abundance differences in the transcripts were clustered by the hierarchical complete linkage clustering method using an uncentered correlation similarity matrix. Prior to the clustering analysis, significantly differentially abundant transcripts (SDATs) generated by pair-wise comparisons were extracted and the abundance data was then pretreated using the standardization tools in Cluster 3.0: (a) log transform data, (b) center genes [mean] and (c) normalize genes. The heat maps were drawn by using the Java Treeview package and the function “sota” of clValid package was used to cluster the SDATs into 16 distinctive clusters with default Euclidean distance and hierarchical clustering method^72^.

### Quantitative real-time PCR (qPCR)

For qPCR analysis, cDNA was synthesized using the PrimeScript^TM^ RT reagent kit (Perfect Real time; Takara, Japan) according to the manufacturer’s protocol with random hexamer primers. The primer pairs for qPCR were designed using the Primer Premier v5.0 software (Premier Biosoft, USA) and listed in Supplementary Table S3. Melting curve analysis was performed for each primer pair prior to further analyses. qPCR reactions were carried out in 96-well plates with CFX96^TM^ Real-Time PCR Detection System (Bio-Rad, USA) using SYBR Premix ExTaq^TM^ (Takara, Japan) quantitation PCR kit. cDNA was diluted five times for qPCR. The reaction mixture consisted of 2 μl diluted cDNA samples, 0.4 μl each of the forward and reverse primers (10 μM), 10 μl real-time master mix and 7.2 μl PCR-grade water in a final volume of 20 μl. The target gene expression levels were normalized using actin gene (TR77358|c0_g1_i4) as internal reference. The relative abundance of transcript levels was calculated relative to the internal reference gene according to the method proposed by Paffl^73^. Three biological replicates and triplicates of each biological replicate were performed for each qPCR analysis and a NTC (non-transcribed control) was also included to confirm correct DNase digestion.

## Electronic supplementary material

Supplementary Information
