# Supplementary material for: Desiccation tolerance in bryophytes: The dehydration and rehydration transcriptomes in the desiccation-tolerant bryophyte Bryum argenteum
Source: Sci Rep. 2017 Aug 8;7:7571. doi: 10.1038/s41598-017-07297-3 (PMC5548717; doi:10.1038/s41598-017-07297-3)
Supplement: Supplementary file 1 — Supplementary Information [file 41598_2017_7297_MOESM1_ESM.pdf]

Supplementary Information

Desiccation Tolerance in Bryophytes: The dehydration and rehydration transcriptomes in the desiccation-tolerant bryophyte *Bryum argenteum*

Bei Gao[1,2], Xiaoshuang Li[1], Daoyuan Zhang[1,*], Yuqing Liang[1,3], Honglan Yang[1], Moxian Chen[2], Yuanming Zhang[1], Jianhua Zhang[2], Andrew J. Wood[4]

[1]Key Laboratory of Biogeography and Bioresource in Arid Land, Xinjiang Institute of Ecology and Geography, Chinese Academy of Sciences, Urumqi 830011, China.

[2]School of Life Sciences and State Key Laboratory of Agrobiotechnology, The Chinese University of Hong Kong, Hong Kong, China.

[3]University of Chinese Academy of Sciences, Beijing 100049, China.

[4]Department of Plant Biology, Southern Illinois University-Carbondale, Carbondale, IL 62901-6509, USA.

* Correspondence should be addressed to D.Z. (email: zhangdy@ms.xjb.ac.cn)

Supplementary Figures and Tables:

Figure S1: Overview of the changes of relative water content (RWC) in cultured *Bryum argenteum* subjected to continuous dehydration and rehydration.

Figure S2: Flow-chart showing steps in *Bryum argenteum* transcriptome assembly and annotation, and downstream transcript clustering and differential expression analysis.

Figure S3: Transcript length distribution of the *de novo* assembled and refined *Bryum argenteum* transcriptomes.

Figure S4: GO category distribution of "Bryum_final" transcriptome among 2nd level GO categories: biological process (BP), molecular function (MF), and cellular component (CC).

Figure S5: BLASTX top-hit species distribution of the Bryum_final transcriptome in NCBI-nr database.

Figure S6: Heatmaps illustrating the expression profiles of the LEA group transcripts upon dehydration and rehydration.

Figure S7: Heatmaps illustrating the expression profiles of the HSP90, HSP20 and HSP33 family transcripts upon dehydration and rehydration.

Table S1: Distribution of percent length coverage for the top matching Swiss-Prot database entries.

Table S2: Percentage of totally and uniquely mapped reads to the Bryum_final reference transcriptome.

Table S3: Selected transcripts and primers used for quantitative real-time PCR (qPCR) analysis.

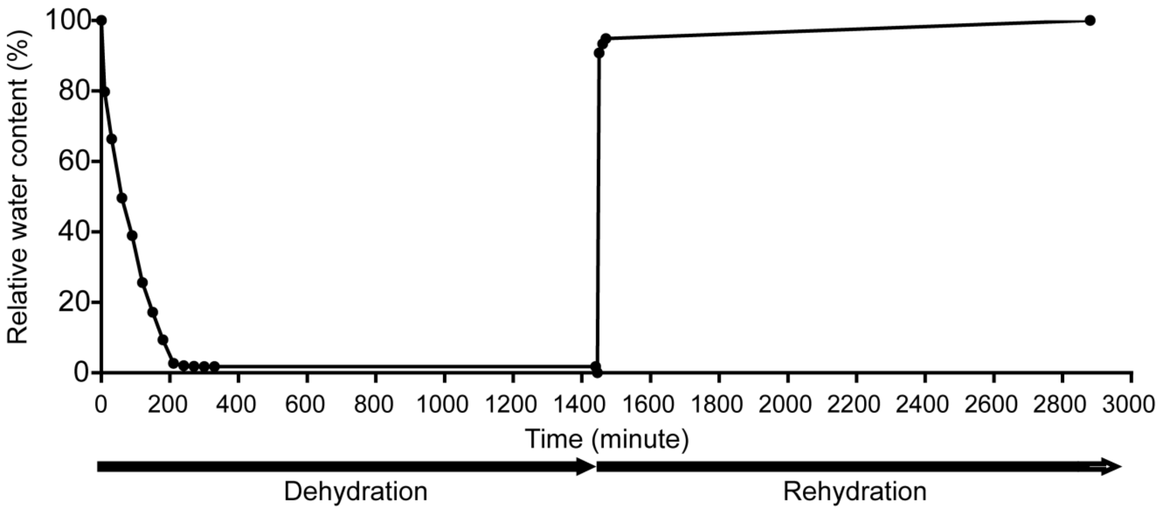

Figure S1. A simple overview of the changes of relative water content in cultured *Bryum argenteum* subjected to continuous dehydration and rehydration.

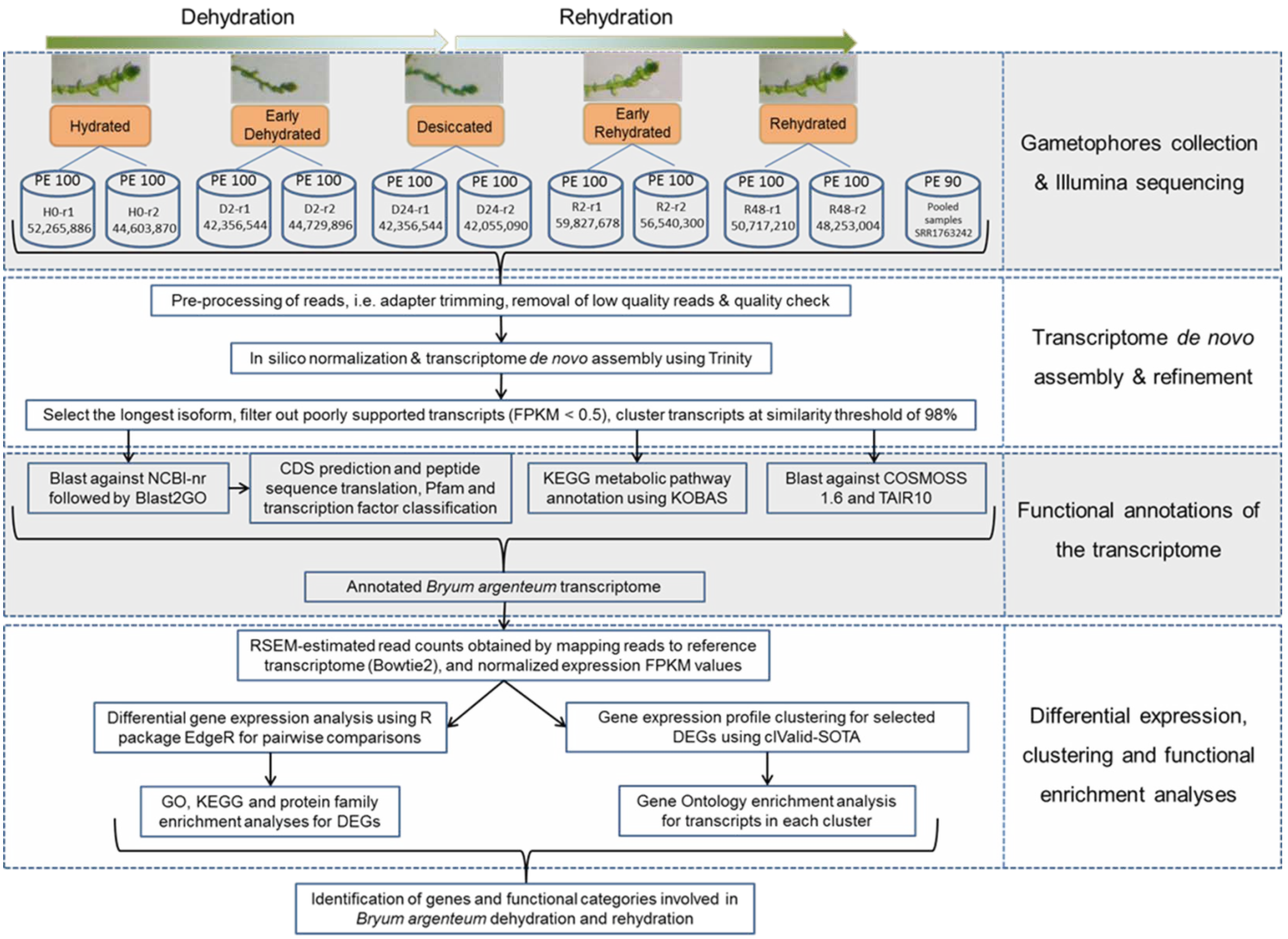

Figure S2. Flow-chart showing steps in *Bryum argenteum* transcriptome assembly and annotation, and downstream transcript clustering and differential expression analysis.

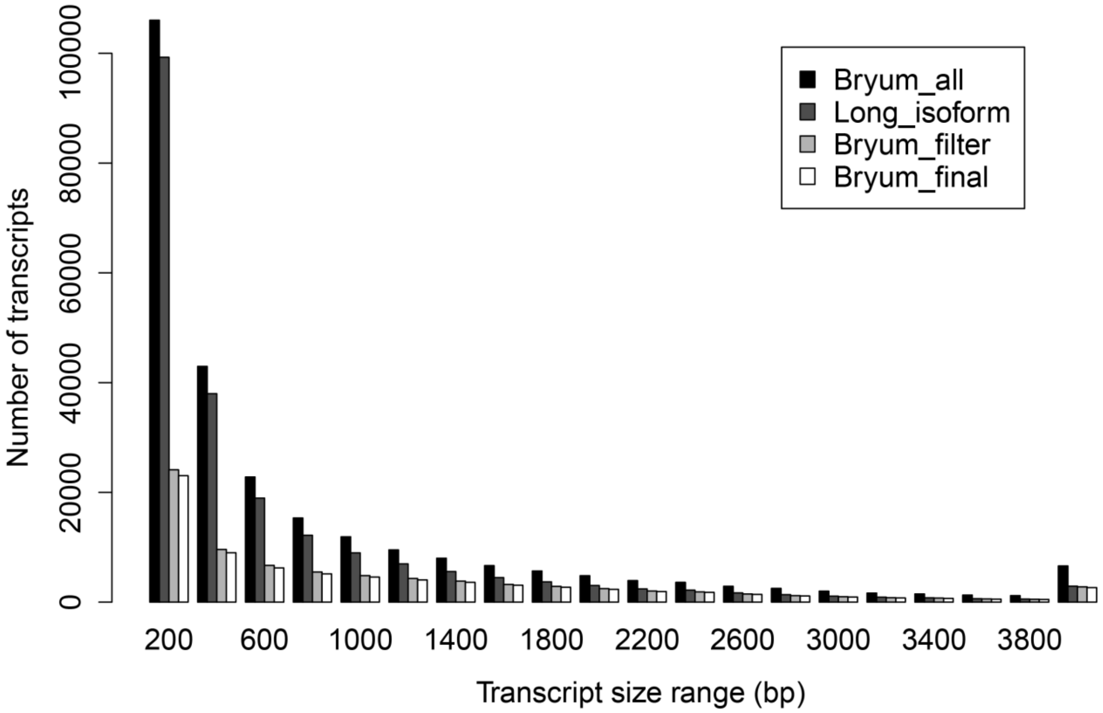

Figure S3. Transcript length distribution of the *de novo* assembled and refined *Bryum argenteum* transcriptomes, demonstrating the extremely high proportion of small-sized transcripts for the initial Bryum_all transcriptome.

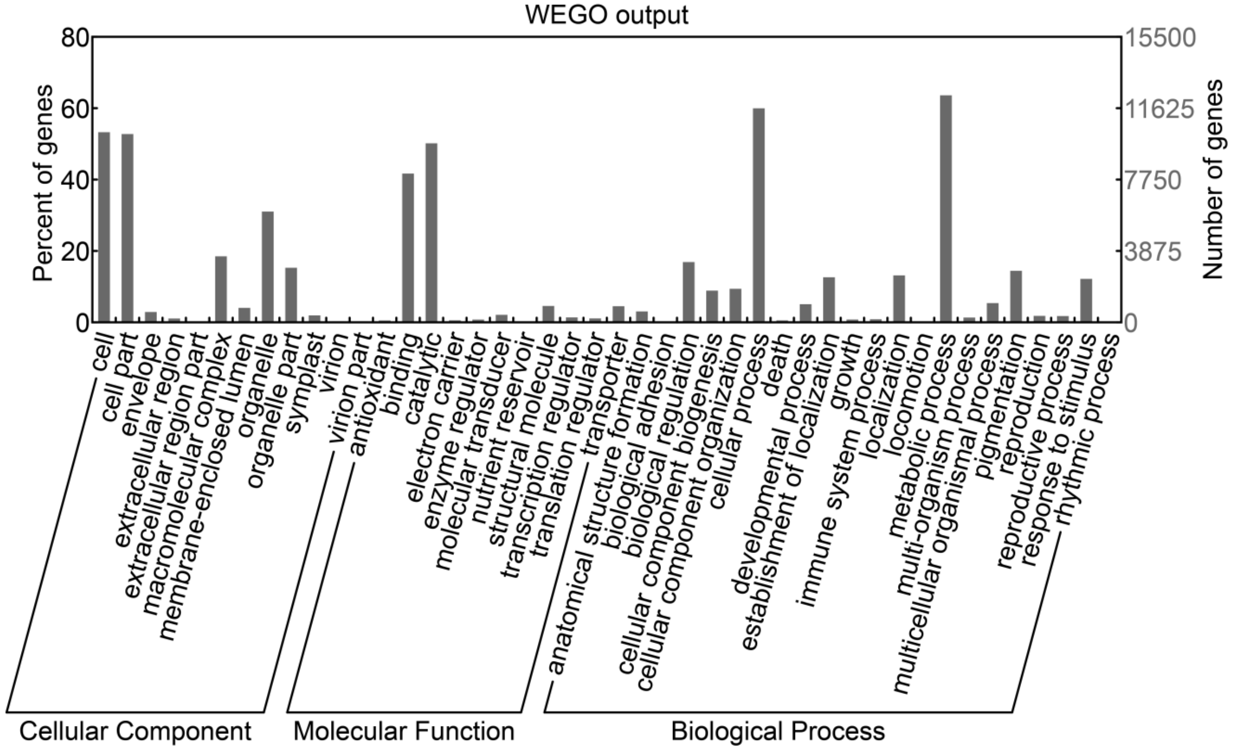

Figure S4. GO category distribution of Bryum_final_transcriptome among level 2 GO categories: biological process (BP), molecular function (MF), and cellular component (CC).

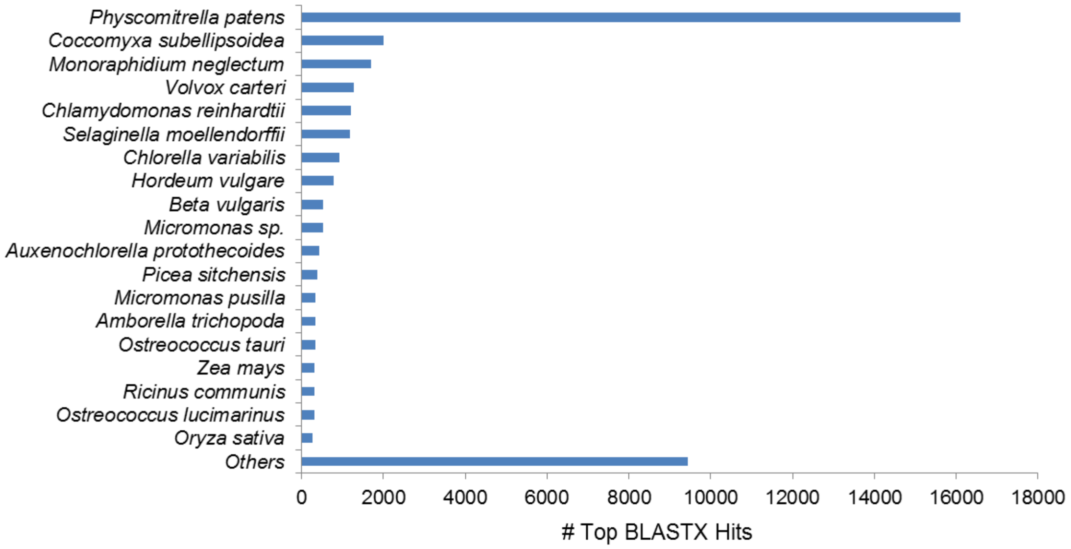

Figure S5. BLASTX top-hit species distribution of the Bryum_final transcriptome in NCBI-nr database.

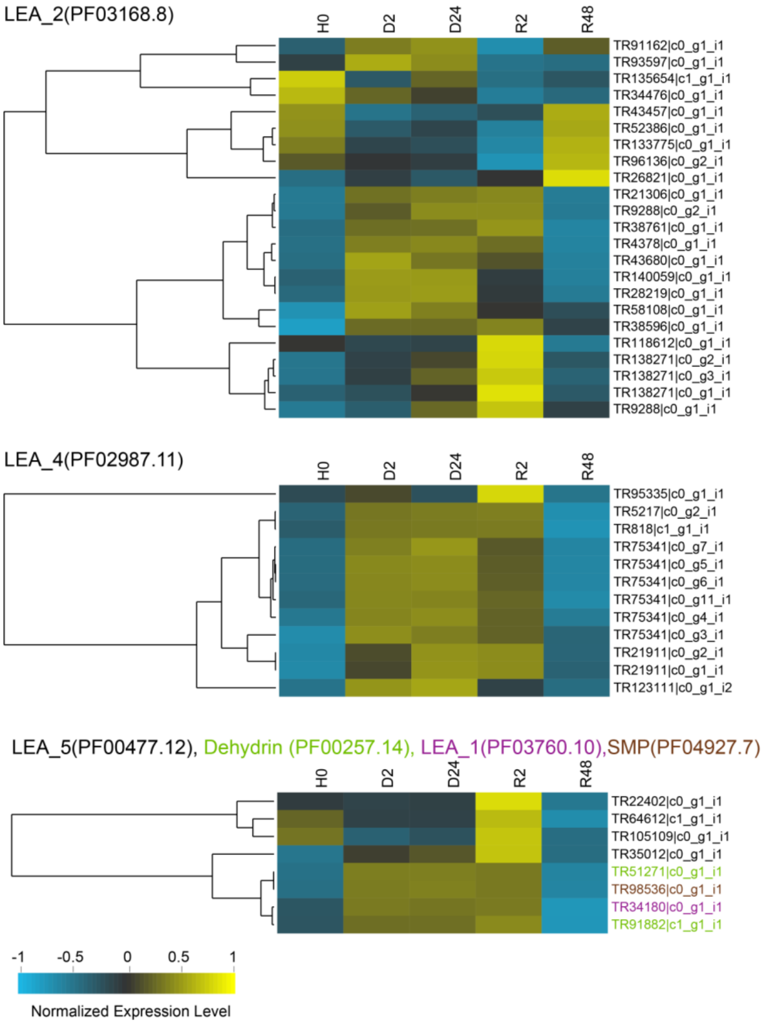

Figure S6. Heatmaps illustrating the expression profiles of the LEA group transcripts upon dehydration and rehydration.

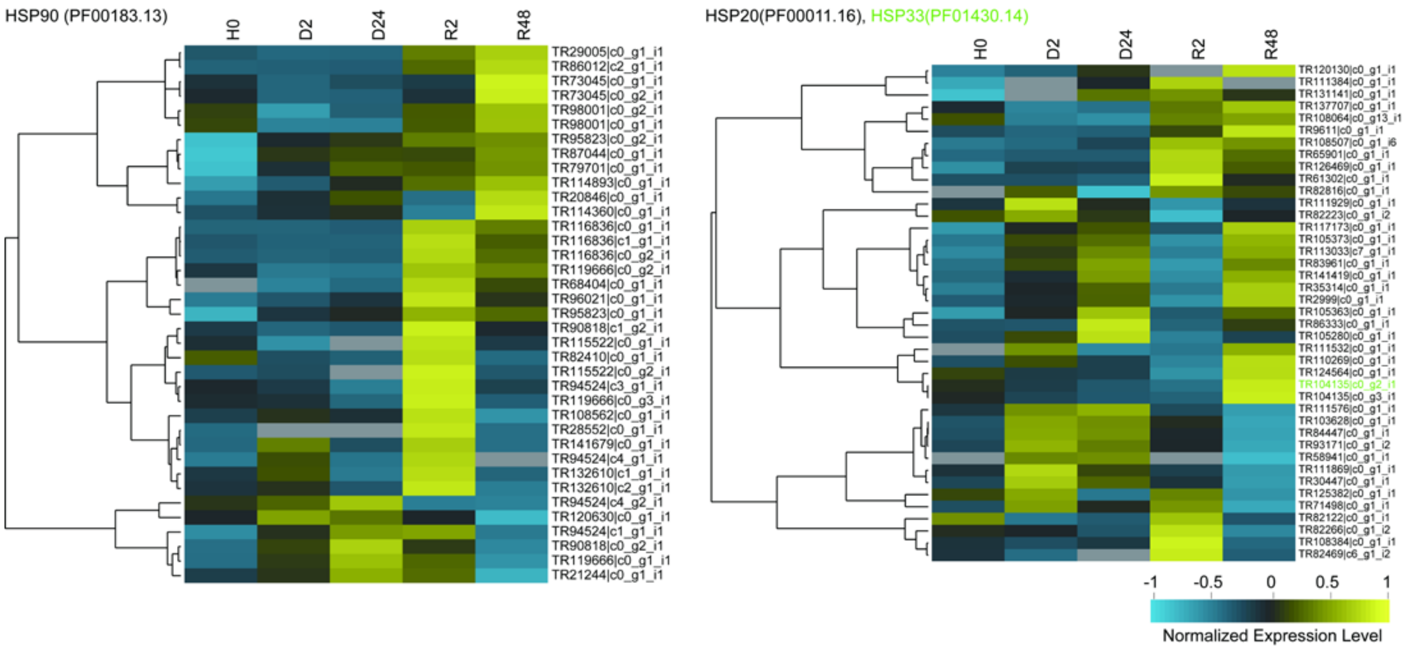

Figure S7. Heatmaps illustrating the expression profiles of the HSP90, HSP20 and HSP33 family transcripts upon dehydration and rehydration.

Table S1. Distribution of percent length coverage for the top matching Swiss-Prot database entries

Percentage length coverage bin	Count in bin	Cumulative count
100	5199	5199
90	2267	7466
80	1423	8889
70	1051	9940
60	942	10882
50	891	11773
40	774	12547
30	684	13231
20	546	13777
10	132	13909

Examples of statements that can be made based on the table above:
- 5199 Swiss-Prot database proteins matches to a Trinity transcript by > 90% alignment coverage.
- 2267 database proteins matches to a Trinity transcript with an alignment coverage between 80% and 90%.
- 7466 database proteins matches to a Trinity transcript by > 80% alignment coverage.

Table S2. Percentage of total and uniquely mapped reads to the Bryum_final reference transcriptome

Sample	Total_mapped_reads (%)	Uniquely_mapped_reads (%)	Multiple_aligned_reads (%)
H0-rep1	84.79	73.71	11.09
H0-rep2	85.12	74.26	10.86
D2-rep1	85.05	74.25	10.80
D2-rep2	84.91	74.18	10.73
D24-rep1	85.17	74.42	10.75
D24-rep2	84.54	73.89	10.65
R2-rep1	83.95	73.18	10.77
R2-rep2	84.23	73.57	10.67
R48-rep1	85.16	76.76	8.41
R48-rep2	85.64	77.38	8.26

Table S3. Primers used for quantitative real-time PCR (qPCR) analysis

Trinity transcript ID	Top hit in *P. patens*	Top hit in *A. thaliana*	Description	Primer pairs
TR89377|c0_g1_i1	Pp1s420_28V6.1 late embryogenesis abundant protein, putative / LEA protein, putative	AT3G53040.1 | late embryogenesis abundant protein, putative / LEA protein, putative	LEA	S: GACAGGGAAACTATCACCGAATC<br>A: GCATCAAGAAGACCACCACCAT
TR72249|c0_g1_i2	Pp1s330_39V6.1 photosystem I light harvesting complex gene 2	AT3G61470.1 | Symbols: LHCA2 | photosystem I light harvesting complex gene 2	LHCA2	S: TTTGTGATGGCTCCACGATA<br>A: CAGACGAAAAGACACTAAGGAT
TR87355|c4_g1_i1	Pp1s93_98V6.1 sucrose synthase 3	AT4G02280.1 | Symbols: SUS3, ATSUS3 | sucrose synthase 3	SuSy	S: CATTAGGGTTGGAGAAAGGC<br>A: CCACGCTGAACACCATAGGA
TR91882|c1_g1_i1	Pp1s442_22V6.2 Dehydrin family protein	No hit found.	Dehydrin	S: CTATGGCGGCGTCAGTGAGC<br>A: CGGAGCGGAAGTCCTGGTAC
TR75290|c0_g1_i4	Pp1s172_69V6.1 ELIP1, ELIP | Chlorophyll A-B binding family protein	AT3G22840.1 | Symbols: ELIP1, ELIP | Chlorophyll A-B binding family protein	ELIP	S: GAGATCGTCCGCAACCAGG<br>A: CAGCTCCATCGAACATCCACA
TR108206|c13_g2_i7	Pp1s97_279V6.1 heat shock cognate protein 70-1	AT5G02500.1 | Symbols: HSC70-1, HSP70-1, AT-HSC70-1, HSC70 | heat shock cognate protein 70-1	HSP70	S: CATCGACGGCACAGACAACG<br>A: CCCTCAACGACTTCTCCACC
TR51243|c0_g1_i1	Pp1s55_256V6.1 Integrase-type DNA-binding superfamily protein	AT4G32800.1 | Integrase-type DNA-binding superfamily protein	ERF	S: TGTTTTCGTATGGGCCTGAA<br>A: ATGTTGCTCTTGCGTGGGAC
TR116182|c0_g1_i2	Pp1s249_84V6.1 heat shock factor 3	AT5G16820.1 | Symbols: HSF3, HSFA1B, ATHSFA1B, ATHSF3 | heat shock factor 3	HSF3	S: AGCAGTCGGCTCAACCTCAG<br>A: TCAGCACATTCTTATCCCTCTTC
TR32094|c0_g1_i1	Pp1s240_91V6.1 Protein kinase superfamily protein	AT4G33950.1 | Symbols: OST1, SNRK2-6, SRK2E, SNRK2.6, P44, ATOST1 | Protein kinase superfamily protein	Protein kinase-SNRK	S: AGTGCGGTTCAAGGAGGTCAT<br>A: AGGAGCAGGGCTTCCATCTAG
TR125288|c0_g1_i5	Pp1s25_29V6.1 zeaxanthin epoxidase (ZEP) (ABA1)	AT5G67030.1 | Symbols: ABA1, LOS6, NPQ2, ATABA1, ZEP, IBS3, ATZEP | zeaxanthin epoxidase (ZEP) (ABA1)	ZEP	S: CCGTCTGTATCTCACTCTGCT<br>A: TTCCCACCGACACTATCAGG
TR94041|c0_g1_i1	Pp1s420_4V6.1 myb domain protein 20	AT1G66230.1 | Symbols: MYB20, AtMYB20 | myb domain protein 20	MYB	S: CTTTGCCGTGGCTCATCTTC<br>A: GCACTTACCGTTGGGCGTCTC
TR77358|c0_g1_i4	Pp1s337_26V6.1 actin 7	AT5G09810.1 | Symbols: ACT7 | actin 7	Actin (internal reference gene)	S: CTCTTAACCCCAAGGCAAACAG<br>A: CTTCATCAGGGCATCAGTCAG
